# Plasma therapy: a novel intervention to improve age-induced decline in deudenal cell proliferation in female rat model

**DOI:** 10.1007/s10522-025-10197-z

**Published:** 2025-02-07

**Authors:** Ender Deniz Asmaz, Taha Ceylani, Aysun İnan Genc, Zeynep Tuğçe Sertkaya, Hikmet Taner Teker

**Affiliations:** 1 Department of Histology and Embryology, Faculty of Medicine, Ankara Medipol University, Ankara, Turkey; 2 Department of Biomedical Engineering Graduate Medical Sciences, Boston University, Boston, MA 02215 USA; 3 Department of Molecular Biology and Genetics, Muş Alparslan University, Muş, Turkey; 4 Department of Biology, Kastamonu University, Kastamonu, Turkey; 5 Department of Physiology, Faculty of Medicine, Ankara Medipol University, Ankara, Turkey; 6 Department of Medical Biology and Genetics, Faculty of Medicine, Ankara Medipol University, Ankara, Turkey

**Keywords:** Cell proliferation, Duodenum, Histomorphology, Plasma treatment

## Abstract

Aging is associated with a disruptive decline in gastrointestinal health leading to decreased duodenal cell proliferation ultimately affecting the digestive and absorptive capacity of intestines in all species. This study investigates the novel application of blood plasma therapy to enhance duodenal cell proliferation associated with aging. In the presented study, the effects of middle aged plasma therapy on the aged rat duodenum were investigated. For this purpose, using a randomized controlled design, Female Wistar rats (aged 12–15 months) (n:7) were treated with heterologus pooled plasma (0.5 mL per day for 30 days, infused intravenously into the tail vein) collected from middle aged (6 months old, n:28) rats during all stages of the estrous cycle. The groups were divided into three as the Experimental group (aged 12–15 months) receiving middle aged plasma, the control group (aged 12–15 months) not receiving treatment, and the middle aged rat (6 months) as the positive control group. At the end of the experiment, each group’s duodenum were collected, fixed, and analyzed using histological techniques for morphometric parameters. Additionally cell proliferation density and proliferation index were determined by proliferating cell nuclear antigen (PCNA). The finding of the study suggests that plasma therapy significantly improves cell proliferation, villus height (µm), crypt depth (µm), total mucosal thickness (µm), the ratio of villus height to crypt depth (µm), and surface absorption area (mm^2^) in the experimental group compared to control. Likewise, we determined that middle aged plasma application supports cell proliferation. However, further research is warranted to explore the underlying mechanisms and potential clinical applications of this innovative approach.

## Introduction

Ageing is an ongoing natural process affecting all species, leading to decreased physiological functions, including gastrointestinal (GIT) health. In humans, age-related GIT declines are greatly associated with a significant impact on longevity and quality of life (Colombino et al. 2021). The duodenum is a vital segment of the small intestine that is critically associated with nutrient absorption and immune-related functions within the body (Bonis et al. 2021). However, the proliferative capacity of duodenal cells gradually declines with age, contributing greatly to GIT dysfunction (Guler et al. 2022). Recent studies suggest that systemic factors present in young blood may influence the decline in tissue regeneration associated with ageing. The plasma therapy is increasingly studied for its regenerative particularly due to its ability to mitigate age-related damage. It has been reported that the plasma is rich in several important growth factors, such as platelet-derived growth factor (PDGF), epidermal growth factor (EGF), basic fibroblast growth factor (b-FGF), vascular endothelial growth factor (VEGF), hepatocyte growth factor (HGF), epidermal growth factor (EGF), transforming growth factor-beta1 (TGF-β1), and basic fibroblast growth factor (b-FGF), that contributes to their biological activity and therapeutic potential (Kaushik and Kumaran 2020; Castellano 2019).

Plasma therapy has been explored for systemic age-related regulatory mechanisms due to its ability to modulate progenitor cell activity (Conboy et al. 2005), for brain health due to improved neurogenesis (Villeda et al. 2011), for cardiovascular ageing and improved endothelial function (Loffredo et al. 2013), age-related obesity, and metabolic syndromes (Zhang et al. 2013) and in improved antioxidant enzyme activities and reduced protein carboxylation in aged rats (Tripathi et al. 2021), indicating potential anti-ageing benefits. Another study shows that plasma treatment can rejuvenate solid organs, extend lifespan, and reduce epigenetic age by up to 30% (Zhang et al. 2021). Research shows that both platelet-rich and young plasma therapy are being explored for GIT diseases including inflammatory bowel disease (IBD), post-surgical interventions, wound healing, enhanced tissue regeneration, and soft tissue injuries by improving cellular proliferation, differentiation, and survival capabilities of the cells (Mourão et al 2018; Shanei et al. 2022). It has also been associated with improved bone regeneration by effectively promoting osteogenic differentiation and angiogenesis (Fujioka-Kobayashi et al. 2020). Additionally, the attenuation of inflammation and oxidative stress in various disease models, using young plasma therapy has led to improved outcomes (Şentürk et al. 2022) and even reversing epigenetic age (Horvath et al. 2024). These efforts have yielded significant findings and have paved the way for more translational research to develop new techniques and procedures.

The intestinal microbiota is influenced by a multitude of factors, including pathological complications, pathogen exposure, hormonal fluctuations, systemic diseases, and the aging process (Gurbanov et al. 2022). Recent advancements in biomedical research have highlighted the potential health benefits of young blood plasma therapy, particularly its regenerative and modulatory effects on various physiological systems. Notably, emerging evidence suggests that plasma therapy may exert significant effects on the intestinal microbiota, which plays a critical role in maintaining intestinal and systemic health. A recent study demonstrated that the application of young blood plasma over a one-month period led to significant improvements in the intestinal microbiota composition of middle-aged rats, further underscoring the therapeutic potential of this approach (Ceylani and Teker 2022).

Although young plasma treatments have been given importance in recent years, young plasma application in the clinic may sometimes not be sufficiently applicable due to the difficulty of the plasma collection process. The fact that young plasma treatment has clinical limitations and that middle-aged plasma is easier and more applicable in the clinic has led us to investigate the effects of middle-aged plasma treatment on aged tissues.

Since the dıgestive system is crucial in maintaining the overall health of an individual, hence sustained mucosa and continuous proliferation and renewal of duodenal are critical to translating the integrity and function of GIT. This study aims to improve nutrient absorption and digestive functions of the GIT by remodelling the intestinal epithelium through enhanced duodenal cell proliferation by using middle-aged blood plasma in aged rats. We further aim to elucidate the underlying mechanism involved in validating the changes induced by middle-aged plasma in improving the villus height, total mucosal thickness, crypt depth, surface absorption area and villus/crypt ratio alongside proliferation index (PI) and proliferation intensity assessed via immunohistochemistry on the ageing digestive system.

## Material methods

### Animal studies

Aged female Wistar rats rats (n:7) (12–15 months) were treated with pooled plasma (intravenously into the tail vein, 0.3 mL per day for 30 days) collected from middle aged (6 months, n:28) rats. After the plasma application, the rats in the experimental group (n:7), control group (n:7) and middle-aged positive control group (MPC) as positive control group (n:7) (6 months) were slightly stunned with ether and sacrificed. Duodenum of sacrificed animals were collected and placed in 10% buffered neutral formalin solution in numbered cassettes and fixed. All animals used in the experiment were kept under standard animal care conditions with free access to water and feed in a temperature-controlled environment. The study was conducted with the approval of the Ethics Committee of the Saki Yenilli Experimental Animal Production and Application Laboratory (approval number: 2022/05/06/21) and was performed with the National Institutes of Health Guide for the Care and Use of Laboratory Animals.

### Plasma collection

Prior to extracting plasma from the experimental cohort, vaginal smears were conducted to ascertain that the 6-month-old subjects, from whom plasma was to be obtained, were in the diestrus, proestrus, estrus, and metestrus phases. Thereafter, seven subjects from each phase were euthanized, and their blood plasma was collected Pooled rat plasma collected by terminal cardiac puncture during euthanasia was prepared from the collected blood with EDTA and centrifuged at 1000 g. Plasma was denaturated by heating at 95 °C for 2–3 min and a short centrifugation at 1000 g and dialyzed using 3.5 kDa D-tube dialyzers (EMD Millipore) in PBS to remove EDTA before administration. Samples were stored at −80 °C until use. (Villeda et al. 2014; Teker et al. 2023).

### Histological and morphometric analyzes of the duodenum

The duodenums of sacrificed animals were taken out approximately 3‒4 cm below the pylorus and placed in 10% buffered neutral formalin solution in numbered cassettes and fixed for 24 h. The duodenums in the fixation solution were passed through a series of increasing degrees of alcohol (50, 70, 80, 90%, absolute), xylol and paraffin, and were blocked with paraffin melting at 58–60 °C. Sections obtained from paraffin blocks were stained with Mallory’s triple staining technique modified by Crossmon for general histological examination (Crossmon 1937). Among the morphometric parameters, villus height (µm), total mucosal thickness (µm), crypt depth (µm), the ratio of villus height to crypt depth (v/c) and surface absorption area (mm^2^) were evaluated. The villus absorptive surface area was calculated using the formula: Villus absorptive surface area = 2π(average villus width/2) × villus height (Yesilbag et al. 2022).

### Immunohistochemical analysis

5 µm thick sections from paraffin blocks were placed on lysine slides, stained with the indirect streptavidin–biotin-peroxidase complex method and examined under a light microscope (Nikon Eclips 80i, Tokyo, Japan).

PCNA primary antibody (sc-7907; Santa Cruz Biotechnology, Inc. Texas) was used in immunohistochemical staining. After the sections were deparaffinized, they were passed through a decreasing alcohol series and Antigen retrieval was performed in a 750 W microwave oven with sodium citrate buffer (1 M, pH 6.1 ABCAM-ab93678) for 3 × 5 min. Following washing with phosphate buffer solution (PBS), the tissues were kept in 3% hydrogen peroxide (Merck 108,600.1000) solution for 10 min to prevent endogenous peroxidase activity. Following washing with PBS, the sections were incubated with the blocking serum (MP7401; ImmPRESS reagent Vector Laboratories, Inc) in the secondary kit for 20 min at room temperature to prevent non-specific protein binding. Then, primary antibody diluted 1:200 as recommended by the manufacturer was dropped onto the sections and kept at + 4 °C overnight. The next day, the sections were incubated with the secondary antibody in the kit for 30 min. In the final stage, 3,3′-diaminobenzidine (DAB-Zymed Laboratories, cat: 00-2020 USA) was used as chromogen and the preparations were counterstained with hematoxylin and covered with entellan.

Proliferative index (PI) was obtained by calculating the ratio of the number of PCNA-positive crypt cells to the total number of crypt cells (Asmaz et al. 2022). It was defined as the average of proliferating cell numbers in 15 randomly selected crypts from the sections (Asmaz and Seyidoglu 2022). In addition, localization and intensity of PCNA expression were also evaluated by two independent observers. In the evaluation made according to the scoring system, 0 means no immune reaction; 1, weak immune reaction; 2, moderate immune reaction; 3, strong immune reaction (Özgüden-Akkoç et al. 2018).

### Statistical analysis

The minimum sample size for the study was calculated as a total of 21 animals, which would provide 80% test power at a 95% confidence level with an effect size f = 0.78 according to the Anova test. IBM SPSS v29 program was used for the analyses of the study. All data are means ± SD: Std. Deviation. Statistical significance between the groups was analyzed by one-way ANOVA test followed by Dunn’s post hoc test. In the analyses, p < 0.05 was considered statistically significant.

## Results

At the end of the experiment, both morphometric and cell proliferation data on the duodenum were evaluated.

### Morphometric evaluation

**Fig. 1:**
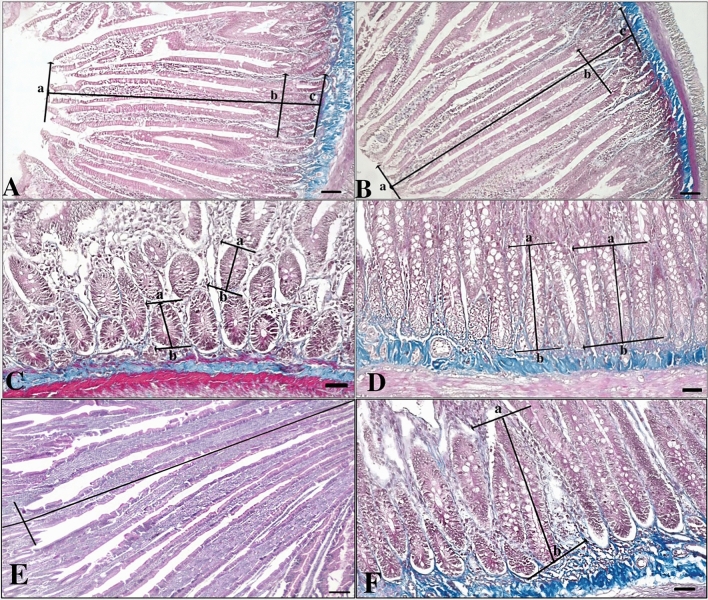
Morphometric evaluation of the duodenum of the control and experimental group. **A**–**C**: Aged group (control), **B**–**D**: Aged + Treatment group (experimental), **E**, **F**: Middle Aged group (Middle-aged positive control MPC) rat duodenum (6 months, old). For **A**, **B**; a, b: villus height, b, c: crypt depth. for **C**, **D**; a, b:crypt depth. **A**–**C**: Bar-100 µm, **B**–**D**: Bar-50 µm

**Fig. 2:**
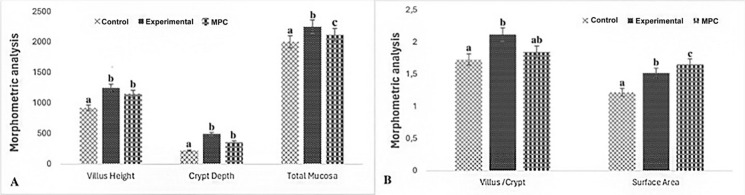
Morphometric measurements in the duodenum after administration of middle aged rat plasma application in aged rat duodenum. **A** Morphometric analysis between villus height, crypt depth, and total mucosal thickness in the Aged group (control), Aged + Treatment group (experimental) and Middle Aged group (Middle-aged positive control MPC) rats. **B** Morphometric analysis between villus/cript ratio and surface absorption area of control and experimental groups. Different letters indicate statistical significance (a, b)

In morphometric analyses, middle-aged plasma treatment applied to aged rats statistically increased the villus height and total mucosal thickness, indicating better utilization from digestion in the duodenum (p < 0.001). In addition, it was determined that the villus/cript ratio, surface absorption area, as well as the crypt depth in the crypts where mitotic cell activity was most intense, increased after treatment (p < 0.001) (Figs. 1, 2) (Table 1).

**Table 1:** Morphometric analysis of the villus height, crypt depth, villus/crypt ratio, total mucosal thickness and villus surface absorption area of the control, experimental and middle age positive control group rats (MPC)

Groups	N	Villus height	Crypt depth	Total mucosa	Villus/crypt	Surface area
Control	15	921,02 ± 16,1^a^	220,5 ± 24,5^a^	2006,7 ± 39,6^a^	1,73 ± 0,11^a^	1,22 ± 0,2^a^
Experimental	15	1248,65 ± 24,5^b^	492,5 ± 21,8^b^	2252,7 ± 38,5^b^	2,12 ± 0,14^b^	1,52 ± 0,01^b^
MPC	15	1150,24 ± 12,1^b^	361,2 ± 19,6^b^	2121 ± 33,2^c^	1,85 ± 0,06^ab^	1,66 ± 0,1^c^
P value		< 0,001	< 0,001	< 0,001	< 0,001	< 0,001

MPC: Young positive control. Different letters in the same column show statistical significance (^a,b,c^)

When the MPC was compared with the experimental and control groups, it was determined that the villus height and crypt depth were higher than the control group, and the total mucosal thickness was higher than the control and lower than the experimental group (p < 0.001). The surface absorption area was higher in the MPC than in all groups (p < 0.001), (Figs. 1, 2) (Table 1).

### Evaluation of cell proliferation intensity and proliferation ındex

**Fig. 3:**
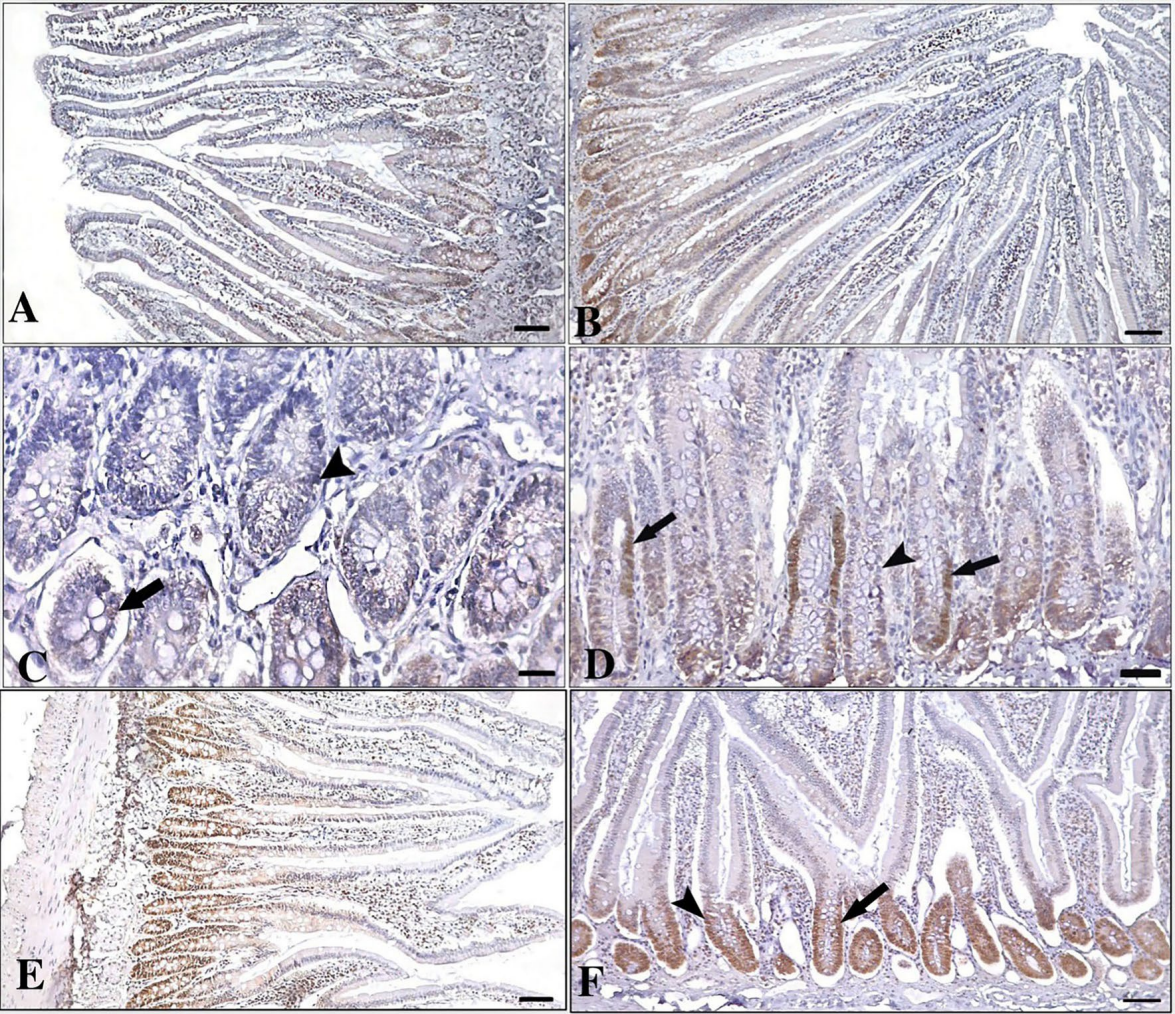
PCNA expression in the duodenum of aged rat treated with middle aged rat plasma application. **A**–**C**: Aged group (control), **B**–**D**: Aged + Treatment group (experimental), **E**, **F**: Middle Aged group (Middle-aged positive control MPC) rat duodenum. arrow: positive immunoreaction, arrowhead: negative PCNA immunoreaction. **A**–**C**: Bar-100 µm, **B**–**D**: Bar-50 µm

**Fig. 4:**
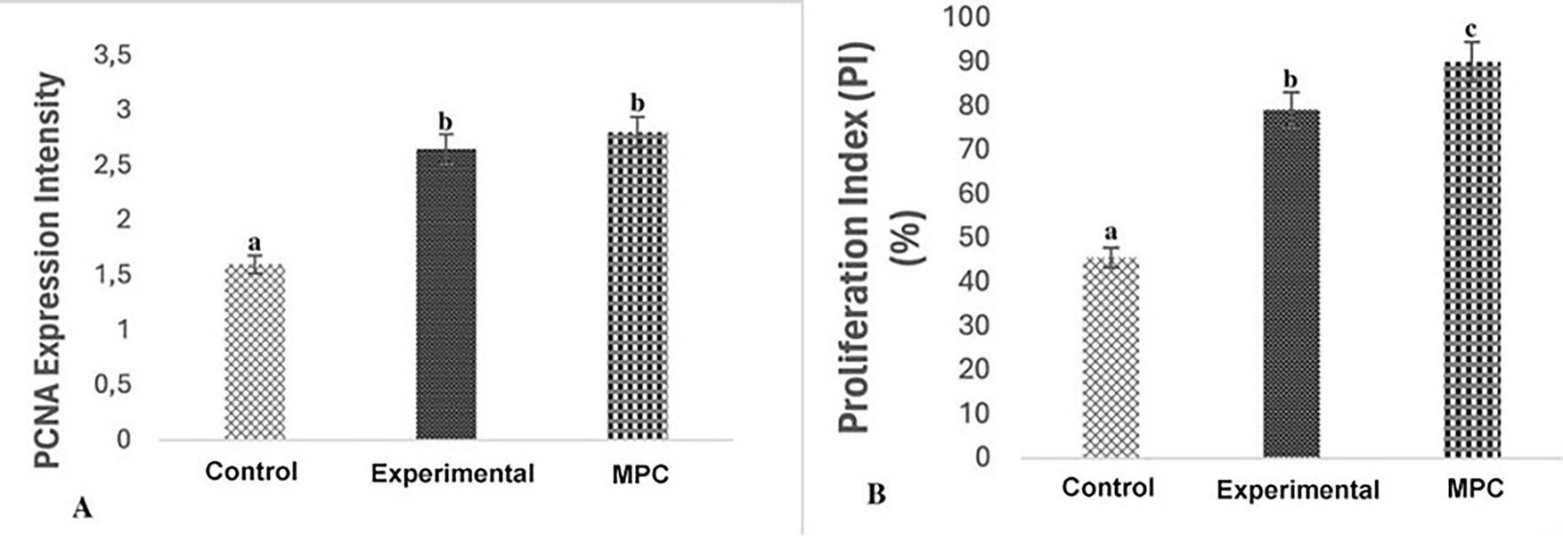
PCNA immunoreactivity on the duodenum of middle aged rat plasma application in aged rat duodenum. **A** PCNA expression severity of Aged (control), Aged + Treatment (experimental) and Middle Aged (Middle-aged positive control MPC) groups. **B** Percentage of PCNA/cyclin positive cells (PI) in the duodenal crypts of control and experimental groups. Different letters indicate statistical significance (a, b)

At the end of the experiment, cell proliferation index and PCNA expression intensity were evaluated in the crypt glands of the duodenum PCNA expression intensity was determined to be weak to moderate in the control group, with cell proliferation still continuing in the aged rats. A moderate to strong cell proliferation intensity was observed in the experimental group of rats. Therefore, the expression intensity in the experimental group increased statistically compared to the control group. Although PCNA expression was most intense in the MPC group, it was statistically determined to be only more intense than in the control group (p < 0.001), (Figs. 3, 4) (Table 2).

**Table 2:** PCNA expression intensity and proliferation index (PI) in control and experimental groups

Groups	N	PCNA expression intensity	Proliferation index (PI)
Control	15	1,60 ± 0,17^a^	45,60 ± 1,82^a^
Experimental	15	2,65 ± 0,19^b^	79,20 ± 1,93^b^
MPC	15	2,80 ± 0,22^b^	90,02 ± 2,02^c^
P value		< 0,001	< 0,001

MPC: Young positive control. Different letters in the same column show statistical significance (^a,b,c^)

The proliferation index determined in crypt cells was found to be higher in the duodenum of aged rats treated with middle aged plasma. This indicates that not only the density of activated crypt cells in terms of expression intensity but also the number of proliferating cells increases after treatment. A higher cell proliferation index was determined in the MPC than in all groups (p < 0.001), (Figs. 3, 4) (Table 2).

## Discussion

Plasma therapy is earning strong recognition for its inherent capacity to improve tissue regeneration, particularly in tissue areas with a high cellular turnover like GIT which is continuously subjected to permanent exposure to microbes, and harsh luminal contents, including gastric enzymes, and acids, making it more prone to injuries. Since, GIT functionality plays a role in humans and animals through various complex mechanisms. Therefore, strategies aimed at regulating the functionality and health of the digestive system are gaining popularity in supporting human health (Celi et al. 2017). Plasma therapy could be considered as landmark due to its ability to modulate the regenerative response in such tissues, with a principal focus on augmenting cellular proliferation.

The digestive system consists of a single cell layer of the epithelial layer, supported by the lamina propria and muscularis mucosa, forming the total tunica mucosa. In the region where nutrient absorption occurs in the epithelial tissue, the presence of villi supports absorption by increasing the surface of the epithelial layer (Helm et al. 2007). Therefore, an increase in the length of these villi structures is important for supporting absorption. Our findings demonstrated a significant increase in villus height, total mucosal thickness, crypt depth, surface absorption area and villus/crypt ratio in the experimental group treated with middle-aged plasma. These morphological changes indicate a substantial improvement in the structural integrity and absorptive capacity of the duodenum. It has been reported that the villus height is directly linked to the mitotic and proliferative activity of stem cells at the base of the crypt glands. These renewed cells migrate upwards from the crypt and renew the intestinal epithelium (Furbeyre et al. 2017). Enhanced villus height, crypt depth, and total mucosal thickness after treatment with middle-aged plasma could be linked with the presence of abundant growth factors in the plasma including PDGF,VEGF,TGF-β, and EGF, all of which are critical for cellular proliferation and tissue repair (Kaushik and Kumaran 2020). PDGF remains a key factor in contributing to the overall thickness of the mucosal by rapid stimulation of the division of fibroblasts and smooth muscle cells (Liu et al. 2023). TGF-β is essential for intestinal mucosal healing, and ΤGF-β modulation of the intestinal epithelium plays a central role in determining susceptibility to injury and is therefore crucial for tissue regeneration. Similarly, Everts et al. (2023) reported that VEGF is pivotal in promoting angiogenesis hence ensuring adequate oxygen and nutrient supply to the regenerating tissue. This is particularly of prime importance in the duodenum, where rapid mucosal turnover is necessary to maintain the epithelial barrier integrity in the intestine. In addition, TGF-β is essential for intestinal mucosal healing, and ΤGF-β modulation of the intestinal epithelium plays a central role in determining susceptibility to injury and is therefore crucial for tissue regeneration (Rowland et al. 2013).

Also Epidermal Growth Factor (EGF) supports the proliferation of intestinal epithelial cells, and signals from various stromal cell populations located beneath the intestinal crypts are important for intestinal epithelial development, daily homeostasis, and tissue regeneration after injury (Abud et al. 2021).

Aging is characterized by a decline in gut epithelial regeneration and barrier integrity, accompanied by significant changes in the gut microbiota. These alterations are linked to increased susceptibility to gastrointestinal disorders, such as irritable bowel syndrome and systemic inflammation (Nicoletti 2015; Parrish 2017). The gut microbiota and the intestinal epithelium are closely intertwined, with antimicrobial peptides and SCFA-producing bacteria playing critical roles in maintaining epithelial integrity (Klement and Pazienza 2019). Our study demonstrated that middle-aged plasma therapy significantly improves histological parameters such as villus height, mucosal thickness, and cell proliferation.

But the total mucosal thickness, which is one of the histomorphological parameters, was determined to be even higher in the experimental group than in the MPC group. Although the parameters of villus height + crypt depth, which constitute the total mucosa, seemed to be numerically higher in the experimental group, no statistical difference was determined between the experimental group and the MPC groups. However, when it came to total mucosa, which is the sum of villus height and crypt depth, we saw that the experimental group was higher than the MPC. Interestingly, the middle-aged plasma given to aged rats increased the total mucosa, which is directly related to nutrient absorption, even compared to the middle-aged rat duodenum. This suggests that the development of histomorphological parameters contained in the treatment given to aged tissue, such as growth factors or cytokines, can rapidly modulate the indicators that are needed due to aging. Although more detailed research is required for this, another interpretation of this situation is that the sample size of the study can be increased.

Additionally, in our prior work, we observed that plasma therapy increased the number of goblets and Paneth cells in aged rats, directly enhancing mucosal barrier integrity, and reduced inflammation by decreasing the expression of TNF-α and COX-2 (Ceylani et al. 2023a). These effects align with shifts in microbiota composition, including increased diversity and normalization of the Firmicutes to Bacteroidetes ratio, which are known to support intestinal barrier repair, epithelial regeneration, and inflammation mitigation (Ceylani et al. 2023b). These findings highlight the synergistic relationship between plasma therapy, microbiota modulation, and epithelial regeneration. By simultaneously improving epithelial integrity and restoring microbial balance, plasma therapy emerges as a promising approach to counteract age-related intestinal decline and promote gut homeostasis.

Our results showed a significant increase in the proliferation index and PCNA expression intensity in treated rats, highlighting the rejuvenating effect of the treatment on cellular regeneration processes. However, a strong expression of PCNA and PI in the MPC group was unsurprising. One of the focuses of our study was to bring the intestinal regeneration and cell proliferation ability of aged rats treated with middle-aged plasma closer to that of young plasma. It has been observed that the applied experimental model and methodology support this. Collectively, these results provide robust evidence supporting the therapeutic potential of middle-aged plasma to counteract age-related degeneration in the gastrointestinal system, thus promoting improved digestive function and nutrient utilization in aged organisms. Our results align with the study conducted by Ceylani et al. (2023a), which demonstrated that the administration of plasma therapy improved biomolecular profiles and reduced inflammation in the ileum and colon tissues of aged rats. The study utilized linear discriminant analysis, Fourier transform infrared (FTIR) spectroscopy, and support vector machine (SVM) techniques to show that plasma therapy restored biomolecular profiles in aged rats to levels similar to those of younger rats. This is consistent with our findings of enhanced cell proliferation suggesting that plasma therapy exerts protective effects on the intestinal tissues of aged rats, thereby improving their histomorphological parameters and cellular regeneration.

Also, It has been suggested that plasma treatment applied to aged rats moderately reduces liver damage against age-related liver damage, suggesting that plasma treatment has beneficial effects on organ damage due to ageing (Liu et al. 2018). Research on blood plasma in ageing-related disorders has shown promising therapeutic potential. Plasma therapy can enhance cognitive functions and improve biological markers in the elderly, while aged plasma may impair younger counterparts. Advanced glycation end-products (AGEs) in plasma are linked to age-related diseases, but their accumulation does not directly correlate with age (Asadipooya and Uy 2019). Advances in plasma proteomics offer insights into organ ageing and disease risk (Argentieri et al. 2024).

Among the animal models used for different treatment methods, mice are used as the primary mammalian model to investigate diseases related to ageing (Kennedy et al 2014; Salpeter et al 2013). It is known that mice can be modelled in transgenic or genome editing approaches thanks to the common pathologies they have with humans (Pan and Finkel 2017; Duran-Ortiz et al. 2021). However, even though some debate about the validity of mouse models (Holtze et al. 2021), the main difference between animal models created to work on ageing studies and the animals selected for many other disease models is that ageing can be easily studied in normal mice or other species (Barghouth et al. 2019; Braga et al. 2021; Brunet-Rossinni 2004). Studies outside the digestive system show that plasma therapy increases synaptic plasticity in aged mice (Villeda et al. 2014) and promotes neurogenesis, thereby alleviating age-related adverse outcomes in the brain (Aicardi 2018; Katsimpardi et al. 2014). The observed increase in cell proliferation in our study, as indicated by elevated PCNA expression in the duodenal crypts, underscores the systemic rejuvenation potential of middle-aged plasma, extending beyond the digestive system to other tissues and organs.

The impact of young plasma on liver health, as explored by Teker et al. (2023), also resonates with our findings. Their study demonstrated that young plasma infusion improved cellular degeneration, hepatic fibrosis, and reduced microvesicular steatosis in aged rats, while aged plasma had adverse effects on young rats’ liver health. The protective and regenerative effects of plasma therapy on liver tissues mirror our observations in the duodenum, suggesting a broad-spectrum anti-ageing effect of young plasma across different organ systems.

In conclusion, our study provides compelling evidence that plasma therapy can significantly enhance cell proliferation and improve histomorphological parameters in the aged duodenum. These findings are consistent with previous research by our team and others, highlighting the systemic rejuvenation potential of plasma therapy. The consistency of these results across different tissues and organ systems highlights the therapeutic promise of plasma therapy as a holistic anti-aging intervention. The increase in cell proliferation after treatment gives us hope that reversible cell regeneration is possible in aged tissues. In addition, we have obtained data indicating that the treatment increases nutrient utilization and facilitates digestion, leads to an improvement in intestinal structure, and increases mucosal integrity. We believe that this treatment method we apply will shed light on the literature in anti-aging treatment studies. Future research should aim to elucidate the underlying molecular mechanisms and explore the clinical applications of middle aged plasma therapy to develop innovative treatments for age-related degeneration, ultimately enhancing health and quality of life during aging.

## Data Availability

No datasets were generated or analysed during the current study.
